# Promising Results from the Use of a Korean Drama to Address Knowledge, Attitudes, and Behaviors on School Bullying and Mental Health among Asian American College-Aged Students

**DOI:** 10.3390/ijerph17051637

**Published:** 2020-03-03

**Authors:** Van My Ta Park, Joyce Suen Diwata, Nolee Win, Vy Ton, Bora Nam, Waleed Rajabally, Vanya C. Jones

**Affiliations:** 1 Department of Community Health Systems, School of Nursing, University of California, San Francisco, CA 94143, USA; bora.nam@ucsf.edu; 2 Department of Public Health and Recreation, San José State University, San Jose, CA 95192, USA; joycesuen24@gmail.com (J.S.D.); nu.hong.vy.ton@gmail.com (V.T.); 3 Department of Nutrition, Food Science, and Packaging, San José State University, San Jose, CA 95192, USA; nolee.win@gmail.com; 4 Department of Sociology, University of California, Merced, Merced, CA 95343, USA; wrajabally@ucmerced.edu; 5 Department of Health, Behavior, and Society, Bloomberg School of Public Health, Johns Hopkins University, Baltimore, MD 21205, USA; vjones@jhu.edu

**Keywords:** mental health, intervention, Asian Americans, school bullying, Korean drama, health education, health disparities, help-seeking

## Abstract

The limited research on bullying, mental health (MH), and help-seeking for Asian American (ASA) college students is concerning due to the public health importance. Korean drama (K-Drama) television shows may be an innovative approach to improve knowledge, attitudes, and behaviors (KAB) on bullying. This study examined whether the KAB about school bullying improved after watching a K-Drama and asked participants about their perspectives of using a K-Drama as an intervention. A convenience sample of college students (*n* = 118) watched a K-Drama portraying school bullying and MH issues. Pre-/post-tests on KAB on bullying were conducted. Interviews (*n* = 16) were used to understand their experiences with K-Dramas. The mean age was 22.1 years (1.6 SD), 83.9% were female, and 77.1% were ASAs. Many reported experiences with anxiety (67.8%), depression (38.1%), and school bullying victim experience (40.8%). Post-test scores revealed significant differences in knowledge by most school bullying variables (e.g., victim; witness) and MH issues. There were varying significant findings in post-test scores in attitudes and behaviors by these variables. Participants reported that they “love” the drama, felt an emotional connection, and thought that K-Dramas can be an educational tool for ASAs. K-Dramas may be an effective population-level tool to improve health outcomes among ASAs.

## 1. Introduction

Research highlights that there is a prevalence of bullying as well as the correlations of experiences of bullying victimization and adverse mental health consequences, such as increased risk for depression and anxiety [1,2]. Bullying is unwanted aggressive behavior that occurs repeatedly in the context of a power differential [3]. Bullying is intricately related to the field of mental health as it is both a cause and a symptom of many psychosocial issues. Perpetrators and victims of bullying both report feelings of serious depression and suicidal thoughts [4]; although victims are significantly more likely to experience depression [5]. Victims of bullying have also been found to have low academic achievement and poor emotional adjustment [6]. Mental health stigma could serve as an obstacle for victims or perpetrators to seek professional mental health, and could also enable victimization by discouraging social support for victims who have manifested mental health issues as a result of bullying. This relays the necessity of our study in focusing on the issue of bullying in relation to mental health stigma.

Mental health stigma is an obstacle for delivering and extending the reach of mental health services to the public [7]. Stigma only discourages individuals from seeking health services, but includes prejudice towards people with mental illness that denies them life opportunities, such as housing and work [8]. 

Furthermore, mental health conditions are significant and preventable [9], but racial/ethnic minority populations such as Asian Americans are less likely to seek help mainly due to cultural barriers (e.g., such as stigma) [10,11,12]. Mental health stigma is further compounded by the notion that Asian American students do not experience bullying and “face few serious challenges” [13]. This is concerning because Asian Americans are the fastest growing racial population in the U.S. [14] and there is a need to better understand the prevalence of bullying among diverse populations as well as provide the culturally appropriate anti-bullying and mental health programs and interventions for such diverse populations. Indeed, the White House Initiative on Asian Americans and Pacific Islanders (AAPI) supports this call for action: “Bullying of AAPI students presents unique circumstances complicated by linguistic, cultural, and religious issues. A wide gap continues to exist between utilization of government remedies by the community and the incidence of bullying and harassment that affects AAPI students” [15].

Moreover, while most studies of bullying deal with children from elementary through high school, very few focus on bullying during college [16,17]. In their literature review on bullying on college campuses, Lund and Ross found the prevalence of bullying was experienced by up to 25% of college students and there is a dearth of research on the influence of race and ethnicity on bullying [18]. Their paper shows that based on the limited research that has been done in this area, there is a myth that bullying ceases after high school.

As the pervasiveness of stigma extends beyond the scope of a mental health practice, advocates have sought to utilize social marketing campaigns such as public service announcements (PSAs) to increase awareness and education regarding mental health. Very few studies have been done to establish a positive relationship between PSAs and its impact on reducing mental health stigma [19]. The primary objective of social marketing bases itself on Fishbein and Ajzens’ theory of reasoned action (TRA), by employing persuasion techniques to direct attitudes of a particular target audience on a particular subject matter [20]. The TRA explains how attitudes, beliefs, and social perception can influence human action [11]. TRA predicts behavior by addressing pre-existing attitudes and intentions. In the case of mental health stigma, mental health is the object at which attitude is directed, and thereafter influences stigma-related behavior.

The literature on social marketing techniques in reducing mental health stigma is limited and provides mixed results with most being delivered through PSAs [21]. While having a broad reach of influence, PSAs only secure brief spans of attention from target audiences [21]. The complexities of mental health stigma necessitate the use of various methods of social marketing that can supplement ongoing efforts. One social marketing medium that has been shown to overcome the limitations that concur with the brevity of PSAs, is television dramas, specifically telenovelas. Characterized under the classification of ‘melodramas’, telenovelas utilize positive character roles to promote specified values and behavior [22]. Also known as entertainment education, this method of social marketing is a nuanced form of behavior intervention and has been used by telenovela creators to promote values, such as female empowerment and tolerance towards race, religion, and sexual orientation [22]. 

Drawing from these previous successes with non-Asian Americans, we theorize that similar results can be reproduced through Korean dramas (K-dramas) with respect to reducing mental health stigma for Asian Americans. K-Drama television shows, which have global popularity [23,24], have often been compared to telenovelas in their themes and plot development [25]. The global popularity of K-dramas lends itself as a useful social marketing tool to promote social and behavioral health. This global phenomenon referred to as K-waves or “Hallyu”, has tripled in size since the early 2000’s with exports of $239 million in 2018 [26]. Korean popular culture has also spread to the United States cultivating a thriving K-drama fan base. KCON, a yearly Korean popular culture convention, drew a combined 110,000 people in Los Angeles and New York [27]. The fascination with Korean popular culture inherently includes an increase in viewership with K-dramas. The U.S. also has a substantial following of K-drama with about 18 million Americans [28]. A substantial portion of viewers is non-Korean speaking in the U.S., and even includes native Spanish speakers [29,30]. Other estimates indicate that there are five to six million users who watch K-dramas on illegal sites in the U.S. [31]. The proliferation in K-drama viewership has even caught the intention of Netflix, which intends to expand into the genre within the coming year [32]. Due to the popularity of K-dramas and its similarity with telenovelas, there is an immense potential for K-dramas to be used as a social marketing intervention.

Given the deleterious consequences of school bullying and poor mental health as well as the limited understanding about school bullying experiences among diverse populations (i.e., Asian Americans) and college students, we conducted a mixed methods study among college-aged students, comprised mostly of Asian Americans with three specific aims. First, we aimed to understand the prevalence of bullying as well as the attributes and characteristics of bullying, depression, and anxiety by gender. Second, we aimed to quantitatively examine the pre- and post-test knowledge, attitudes, and behaviors (KAB) of school bullying, mental health, and help-seeking. Third, we aimed to qualitatively understand participants’ experiences and perspectives of utilizing a K-drama show to help address school bullying, mental health, and help-seeking.

## 2. Materials and Methods

### 2.1. Participants

This study included participants who were enrolled in a large urban university in the San Francisco Bay Area in California, United States of America, at the time of the study. Recruitment methods included flyers and classroom announcements. Inclusion criteria for the study required participants (a) being able to speak, read, and write English; (b) were 18 to 25 years old, and (c) have not watched previously the Korean drama titled, “*School 2013”, the K-drama used for the intervention, described below.*

A total of 130 participants completed the quantitative questionnaires; however, 12 of the 130 participants reported that they were “unsure” about their victimization experience with school bullying. These 12 participants were subsequently excluded from the analyses as our outcome of interest pertains to victimization experience with school bullying. Moreover, the small number of 12 makes it difficult to conduct meaningful analyses. The sample for the qualitative analysis for the current study is 118.

### 2.2. Intervention: Korean Drama Television Show

The study’s intervention is a K-drama television show, called *School 2013,* which portrayed the struggles and problems that youth experience, such as school bullying and mental health issues. *School 2013* included 16 episodes that are each one hour in length. Similar to many other K-dramas, *School 2013* is free and available online. 

### 2.3. Design and Methods

This mixed methods study that employed a pre- and post-test design. Participants signed an online Qualtrics informed consent form prior to completing the online surveys about their demographic background, experiences with school bullying, mental health, and a pre-test on their knowledge, attitudes, and behaviors on school bullying. After the participants completed these online surveys, the participants were instructed to watch *School 2013.* Finally, after they finished watching *School 2013,* the participants completed the online post-test survey. To ensure that the K-drama viewing group participants watched the K-drama, they were asked two questions drawn from the plot of the K-drama. The participants in the K-drama viewing group, who incorrectly answered both questions were not given the post-test and are not included in this analysis. This process was used to determine that this pilot evaluation was comparing participants who saw all 16 episodes of K-drama and those who did not see it. An online list of local mental health resources was provided to the participants after they had completed the post-test survey. Extra credit points were given for their study participation. There was no attrition for this study.

After the completion of the quantitative study, a recruitment email was sent to the 118 participants inviting them take part in semi-structured interviews. Sixteen interviews were conducted with a convenience sample of all who responded to be interviewed after obtaining a signed informed consent. Two trained research assistants administered the interviews. Audio-recorded interviews took place at a private room at the university. Each participant received a $5 gift card or a movie ticket for their study participation.

### 2.4. Measures

Participants provided self-reported data for the following questionnaires:

Socio-demographic questionnaire. Participants were asked questions about their age, gender, ethnicity, nativity, language(s) other than English that they can speak/read/write and their fluency level, marital status, educational level, employment status, occupation, experiences with watching K-drama, and experiences with school bullying. 

Mental Health and School Bullying Questionnaire. Participants were asked about their lifetime experiences with mental health (depression and anxiety) and school bullying (as a victim, perpetrator, and witness).

Depressive symptoms. The Center for Epidemiologic Studies-Depression (CES-D) scale is a 20-item, self-reported scale that assesses the presence of depressive symptoms in the past week [33]. The items provide an index of cognitive, affective, and behavioral symptoms with possible responses to each ranging from 0 (none of the time) to 3 (most or all of the time) based on frequency of occurrence. The total scores can range from 0 to 60, with higher scores indicating more depression symptoms and at a greater occurrence. This study used the cut-score of 16 or greater as an indication of depression [34,35]. The CES-D has been used and validated with Asian Americans [36,37,38]. Compared with the original internal consistency estimate (based on Cronbach’s alpha) reported for the CES-D of 0.85 [33], the estimate yielded for this study was 0.90.

Pre- and post-test survey on knowledge, attitudes, and behaviors (KAB) on mental health and school bullying (Appendix A). The pre-test and post-test surveys included a total of 40 items including items on their KAB on mental health and school bullying. The items were scored on a five-point Likert scale from “strongly disagree” (“1”) to “strongly agree” (“5”). Since there are limited school bullying surveys for college-aged students, the study generated survey items guided from the following validated questionnaires or developed items for the purpose of this study:

(1) Authoritative School Climate Survey (ASCS) by Dewey Cornell et al. [39,40]. The purpose of the ASCS is to assess school climate and bullying in secondary school settings. Reliability and validity scores vary depending on the scale (e.g., bullying victimization; student support) but are considered to have good reliability and validity scores across scales. Eighteen items were adapted/guided from the 84 ASCS items; 

(2) Measuring Bullying Victimization, Perpetration, and Bystander Experiences: A Compendium of Assessment Tools—Centers for Disease Control and Prevention (CDC) [41]—22 items were drawn from this compendium of tools which included 33 bullying reliable and validated measures that were conducted among 12–20 years old students. *Note: both the Authoritative School Climate Survey and CDC survey were used to guide the development of three survey items*; 

(3) Revised Pro-Victim Scale by Rigby (original by Rigby and Slee) [42,43]—one item (“Kids who get picked on a lot usually deserve it”) from the scale’s 20 items was adapted from this scale to state, “Students that are bullied usually deserve it” for this study. The scale has high reliability and validity; 

(4) Two items, “You have prior knowledge on school bullying,” and “You are aware of the resources offered at your university regarding school bullying,” were developed for the purpose of this study.

### 2.5. Semi-Structure Interview Guide

Participants were asked about their experience with watching *School 2013,* experiences with K-dramas overall, and perspectives about Korean dramas’ potential utility to address school bullying, mental health, help-seeking, and other public health issues. Examples of specific questions asked included:

1. What do you think about Korean dramas (K-dramas)?
2. What did you think about *School 2013*?
3. We are evaluating the use of K-dramas to help address mental health issues and help-seeking among young adults. What are your thoughts about this? Do you think K-dramas can be a good way to do this?
4. Hypothetically, if we (the research team) were to be able to work with a K-drama production company, do you have suggestions on how K-dramas can help address mental health issues and help-seeking among young adults? If yes, what are your suggestions?
5. Do you think K-dramas can be used to address other health issues? If yes, what are some examples?

### 2.6. Data Analysis

Statistical analyses were performed using STATA 15.1 software (College Station, TX, USA) [44]. Univariate analysis was conducted by examining the distribution, central tendency, and the dispersion of each variable. Chi-square goodness of fit test, Fisher’s exact test (categorical variables), and t-test (age as a continuous variable) were employed to examine the associations between the socio-demographic variables and victim of school bullying experience (see Table 1) as well as mental health and school bullying by gender (see Table 2). The primary outcome was a composite score of knowledge (12 items), attitude (20 items), and behavior (8 items) (see Table 3 and Table 4). The KAB composite score of each participant was identified as an overall mean score across all items. Eight KAB items were reverse coded (see Appendix A). A paired t-test was applied to test for differences in the means of the KAB pretest and posttest scores among both those who reported experience of being a school bullying victim as well as without this experience. The level of statistical significance was *p* < 0.05.

Interview data were transcribed into a word document. Content analysis [45], using a directed content analysis approach [46], was employed in this evaluation. The principal investigator, who designed the semi-structured questionnaire, created a coding dictionary based on the questions a priori. Then, two raters independently utilized the coding dictionary as a guide to analyze the qualitative data. Upon comparison of the analyses, the raters resolved any discrepancies in the emergent themes, which were pieced together to form a comprehensive understanding of the participants’ perspectives [47]. Data saturation was reached for the prevalent themes.

### 2.7. Human Subjects Protection

This research was approved by the San José State University Institutional Review Board. Informed consent was obtained from the participants prior to study participation.

## 3. Results

### 3.1. Sample Socio-Demographic Characteristics, by Victims of School Bullying Experience (Table 1)

Overall, two out of five participants (40.8%) reported that they had been victims of school bullying. The majority were female (83.9%) and were single or not married (97.5%). Many were born in the United States (U.S.) (72.0%). About seven out of ten participants (71.2%) were working full- or part-time. In terms of ethnicity, 65.3% reported being non-Hispanic, 17.8% Hispanic, and 16.9% unknown. In terms of race, 77.1% reported being Asian or Asian American, 5.9% other (Black or African American (*n* = 1); White or Caucasian (*n* = 2); and other, not specified (*n* = 4)), and 17.0% unknown. Most reported fluency in another language. The mean age of the participants was 22.1 (SD ± 1.6) years. There were no significant differences in socio-demographic characteristics by victim of school bullying experience, though males reported a higher proportion of experience as a victim of school bullying compared to females.

### 3.2. School-Bullying Experience, by Gender (Table 2)

Overall, 38.1% reported that they had experience with depression, and there were no significant differences by gender. A great proportion had a CES-D score of 16 or greater (43.2%), which suggests possible depression, and there were significant differences by gender with more than twice the proportion of females (47.5%) having possible depression compared to males (21.1%) (*p* = 0.043). Many (67.8%) participants reported that they had experience with anxiety in their lifetime, but there were no significant differences by gender.

About one in ten participants (11.9%) said they were perpetrators of school bullying in their lifetime, and there were significant differences by gender (26.3% of males vs. 9.1% of females, *p* = 0.008). About 76.3% said they have witnessed school bullying, but there were no significant differences by gender.

### 3.3. Comparison of Pre- and Post-Test Composite Mean Scores on Knowledge, Attitudes, and Behaviors (KAB) of School-Bullying Experiences (Table 3)

Table 3 shows that there were significant differences in the pre- and post-test composite mean scores on knowledge among both those who reported experience of being a victim of school bullying (*p* = 0.006) as well as without this experience (*p* = 0.004). There were no significant differences in the pre- and post-test composite mean scores on attitudes among both those who reported experience of being a victim of school bullying *p* = 0.127 as well as without this experience (*p* = 0.078). There were significant differences in the pre- and post-test composite mean scores on behaviors among those who reported experience of being a victim of school bullying (*p* = 0.003) but not by those without this experience (*p* = 0.738). 

Significant differences in the pre- and post-test composite mean scores on knowledge were found among both those who witnessed school bullying (*p* = 0.003), those who did not witness school bullying (*p* = 0.026). There were significant differences in post-tests on attitudes among those who had no experience witnessing school bullying (*p* = 0.012) but not by those with this experience (*p* = 0.126). There were no significant differences in the behaviors post-test scores for those who witnessed or did not witness school bullying.

There were no significant differences in the pre- and post-test composite mean scores on knowledge and attitudes among those who reported experience of being a perpetrator of school bullying. For those without experience of being a perpetrator of school bullying, there were significant differences in the pre- and post-test composite mean scores on knowledge (*p* < 0.001) and attitudes (*p* < 0.001). There were no significant differences in the behaviors scores for those who were or were not perpetrators of school bullying.

### 3.4. Comparison of Pre- and Post-Test Composite Mean Scores on KAB of Mental Health Issues (Table 4)

Table 4 shows that there were significant differences in the pre- and post-test composite mean scores on knowledge among both those who had experience with depression (*p* < 0.001) as well as without depression (*p* = 0.043). There were no significant differences in the pre- and post-test composite mean scores on attitudes among both those who had experience with depression as well as without depression. There were significant differences in the pre- and post-test composite mean scores on behaviors among those who had experience with depression (*p* < 0.001) but not by those without depression. 

Presence of depressive symptoms showed significant differences in the pre- and post-test composite mean scores on knowledge (*p* = 0,006), attitudes (*p* = 0.042), and behaviors (*p* = 0.015). There were significant differences in the pre- and post-test composite mean scores on knowledge (*p* = 0.004) but no differences in the attitudes and behaviors scores among those without depressive symptoms. 

There were significant differences in the pre- and post-test composite mean scores on knowledge (*p* < 0.001), attitudes (*p* = 0.007), and behaviors (*p* = 0.005) among those who had experience with anxiety but not by those without anxiety.

### 3.5. Qualitative Themes

Four themes emerged through the analysis: (1) experience with watching *School 2013*; (2) perception of K-Drama; (3) efficacy of the use of K-Drama; and (4) suggestions on addressing mental health issues in K-Dramas. 

Theme 1: Experience with watching Korean drama (School 2013). Most participants reported that they had positive experiences watching *School 2013*, and others said they had neutral experiences (there were no participants who reported negative experiences). Participants mentioned that they were unaware of school bullying occurring in their school, especially if they were not the victims or if they did not know the victims. After watching *School 2013*, participants realized that school bullying may occur more often than they thought, and they felt sympathetic towards those who experienced school bullying.

Participant: *“Yeah, I think it was a positive experience because when you watch Korean dramas you learn a lot from it like how melodramas you are crying and you are happy you want that experience and you want it. But for this drama you see school bullying in action you don’t hear it. But seeing it portray makes other people know about it more than to just hear it.”*

Participant: *“I think this specific drama could give people more insight and make them more aware of what is going on around the high school environment because some people are not as aware than those who are going through school bullying or pressure from school. I think it can help with mental health issues since it gives them insight.”*

Theme 2: Perception of K-Drama. Perceptions of K-Drama varied depending on whether they had ever watched K-Drama shows before or if this was their first time watching a K-drama show. Many participants viewed K-Drama as entertaining and enjoyed watching K-Dramas. Participants also expressed that the plots may be dramatic and sometimes predictable, but understood that such plots were necessary in order to grasp the viewers’ attention.

Participant: *“I would say certain scenes are exaggerated. Like the bullying aspect would be exaggerated in order to make its point to let the viewer know so that the viewer can feel the impact of what the character is going through.”*

Many participants said that they found the K-drama show relatable, felt an emotional connection to the K-Drama such as remembering past personal experiences, and elicited emotional reactions to school bullying.

Participant: *“There are a few characters that caught my attention regards to personal experience and social issues, especially in Asian culture.”*

Participant: *“I feel like you get more insight about other topics such as relationships. For this specific Korean drama I learned a lot on school bullying and the pressure you get from your parents about school.”*

Participant: *“Because it is so, so real and sometimes when you watch it, it’s so realistic that you sometimes get sucked into the movie and you feel like you’re in there so sometimes you would ask yourself, what I would do and how I would do this differently. And, I think that’s what does it because it’s so surreal sometimes.”*

Participant: *“I do think it is relatable because not specifically a gang bullying me but having high school friends bullying and having my mom pressure me for school. My mom used to hit me when I failed a test or something.”*

Participant: *““The teachers and professors in the U.S. sometimes don’t have much compassion towards students. In Asian countries, teachers have a lot of respect. Maybe it has to do with the education system in the U.S.”*

Participant: *“In the beginning it made me mad that they were bullying the quiet one. And it’s always the quiet one. It happens in real life too that they pick on the smallest or quietest one. It made me feel bad inside cause I feel like this Korean drama is supposed to make other people feel like school bullying does happen and that bullying does affect individuals and impacts someone’s life.”*

Participant: *“My first thought was not on the drama but the bullying itself, as in, these kids are really strong at heart and even though they face problems outside of school, they kept it to themselves, the fight for it. It’s not just about being bully as we see in the movie, it’s about their personal and individual thought about the whole process. They fought like family problems and stress and that’s a lot.”*

After watching the K-Drama show, some said that their perspectives on Korean culture and as well as different K-Drama character personalities had broadened.

Participant: *“It shows you the broader perspective of how students are being treated at school and how they don’t tell their parents or act out because their parents are never there for them.”*

Theme 3: Efficacy of the use of K-Drama. Participants reported that K-Dramas can have a population level change because it can visually portray how school bullying can affect individuals’ life in school and at home. Due to the popularity of K-Drama among a young adult audience, participants also felt that K-Dramas can be used as a visual aid to influence viewers’ emotions and behavior. Furthermore, participants also said that K-Dramas contain diverse genres (e.g., school life; family life) which creates a sense of commonality among young adults.

Participant: *“I think they can help everyone. Most people who watch it have a better view of how to treat others. K-drama is like a great way to help educate students how to treat their classmates.”*

Participant: *“Some people don’t understand what children do in school and some students do not tell exactly what happens to them with their parents and they keep it a secret. Now the K-drama can give them a little information about what happens to the children in that situation.”*

Participants also mentioned that K-Dramas might have the potential to promote help-seeking behaviors by educating viewers on how they can help victims of school bullying and where they can get help.

Participant: *“I feel like in the Korean dramas they can actually promote actual locations to get help for mental health.”*

Participant: *“I watch a lot of dramas and my attitude to other students are like more friendly. Most students I see who watch Korean drama are really friendly. It’s like they got taught to how to behave around their classmates because they watch it since they were in high school—when they get to college they’re really friendly. I believe that it’s a way to educate other students. Before I wouldn’t step up to help anyone when I see someone get bullied but after watching drama I feel like you need to help them. You can’t do much but help them to ask the professor that they’re in that kind of situation and what can they do to help. ”*

Theme 4: Suggestions on addressing mental health issues in K-dramas. Some participants said that providing resources such as suicide hotlines in the K-dramas would help viewers feel supported if they are going through distress.

Participant: *“Putting a website or a link or hotline to contact people if they are or happening to be going through the same thing. They could reach out to people that need help or are too scared to like contact anyone else. It can help those people suffering those issues and other mental health issues.”*

In addition, participants said that K-dramas may play an important role in stress in helping to educate viewers on prevention, especially if they can relate to the topic.

Participant: *“I feel like stress can be one of them, college students are stressed and I think they can relate to a drama on stress. I think it will be good to show on how we can prevent them or reduce it as much as possible.”*

Some participants addressed that *School 2013* portrays certain emotional strain such as stress, which can lead to anxiety, anger, and depression. However, participants felt that mental health issues are stigmatized topics, but K-dramas can help to shed light on these topics.

Participant: *“I don’t think depression has been shown at all or publicized at all even though it’s super problematic in Korea and I think that’s something that should be touched.”*

Participant: *“I feel that addressing depression in one of the K-dramas would be kind of helpful because it shows the audience what it is like to be suffering depression in their shoes.”*

Participant: *“It’s really important for college communities, a lot of students do feel afraid and they hear that word (counseling) and they feel like, ‘Oh no, I don’t need counseling,’ but there’s more to it than just talking and it’s more than that. Same with therapy, it just scares people away and I feel using Korean drama will help.”*

## 4. Discussion

### 4.1. Summary of Findings

This study attempts to determine if K-dramas could be a useful tool to address bullying for college students. Specifically, the study examined the changes in the KAB scores after watching a K-drama on school bullying (quantitative study with 118 participants) and asked 16 participants about their perspectives and experiences watching the K-drama show. Our findings reveal that bullying victimization experience is common among our sample of mostly Asian American college students. Specifically, about 41% of the study participants reported experience with being a victim of school bullying, which is much higher than that reported in a review paper on victimization experience among college students (up to 25%) [18]. Additionally, students also reported high rates of anxiety and/or depression, all are risk factors and consequences of bullying. Given the significance of higher mental health issues and bullying, and the large proportion of our sample that has both, these findings support a population approach to bullying prevention and mental health. 

This study found that K-dramas were most influential for increasing knowledge changes for those with and without victim of school bullying experience but there were no significant differences found for attitudes. The study also found that the post-test scores for behaviors changed significantly among those with victim of school bullying experience, but not for those without such experience. The post-test scores for knowledge were significantly changed among both those who witnessed school bullying and who did not. Only those who did not witness school bullying showed significant changes in post-test scores in attitudes. The study also found that the post-test scores for knowledge and attitudes changed significantly among those who were not a perpetrator of school bullying, but not for those with such experience. However, there was no significant change among behavior scores. Though it is challenging to compare these study findings with other studies’ findings (i.e., social marketing television intervention studies on bullying), the literature on other forms of social marketing (e.g., social media outreach for bullying) [48] highlights an opportunity for K-dramas to be an influential social marketing tool for bullying. 

This study also found that K-dramas were influential for increasing knowledge, attitude, and behavior changes for those with depressive symptoms as well as those who had experience with depression or anxiety. However, there were no significant changes for those without such experience. The study findings are also similar to those reported in other K-drama research that reported significant increases on participants’ post-test knowledge, attitudes, and behaviors about precision mental health, which includes aspects of mental health and help-seeking [49]. 

As suggested by Bolton and Flemmington (1996) [16], this study explored the perceptions of K-dramas for bullying prevention among participants through quantitative investigation. Participants felt that the use of K-dramas is innovative, entertaining, and culturally acceptable as supported by the qualitative findings. The first theme underscored the positive experience of watching K-dramas and that knowledge can be gained during the viewing. The use of K-dramas with a college population of young adults has the potential to elicit an emotional response, also highlighted by this theme. Moreover, participants talked more about their personal awareness of bullying after watching the show. They were able to connect their prior experiences to the scenarios in the K-drama. Additional qualitative themes suggest that K-dramas are helpful for providing additional community context for how to address bullying and to seek mental health services. Lastly, as previously identified in a participant quote, future development of K-dramas should provide local information for mental health services to help facilitate help-seeking behavior. Again, these qualitative findings are consistent with prior K-drama precision mental health research in that the use of K-dramas as a health educational tool is innovative and may be widely used with Asians in the U.S. and globally [49].

### 4.2. Strengths and Limitations

A noted strength of this study is the innovation of using a popular cultural phenomenon (K-dramas) as a bullying and mental health intervention. In addition, this was a low-cost intervention study because the K-drama show was available and accessible online for all viewers at no cost, making this a feasible study to conduct. Furthermore, this study contributes to the limited understanding about school bullying victimization experience and mental health among an understudied demographic [18] (i.e., Asian American college students). Also, given the anonymity of the online surveys, social desirability bias might have been minimized. Study limitations include the use of a convenience sample and self-reported data. Due to resource constraints, the study did not conduct follow-up post-tests to investigate change on the participants’ knowledge, attitudes, and behaviors over time. Also, the study did not ask participants if they liked the K-drama, changes they would make to the K-drama, or about their specific racial subgroup (e.g., Korean Americans; Chinese Americans); thus, future research should collect this data to examine its utility among different Asian subgroups. The study did not include a control group to compare the change in KAB scores over time with the intervention group; hence, future research may consider adding a control group to their study design.

While the K-drama discussed in this study, showing bullying situations, can cause a stressful response or be a stimulus for stress based on experiences, these dramas are also intentionally providing solutions to addressing bullying. Thus, instead of only producing an emotional response, as entertainment, the approach to using K-dramas as a public health tool, requires healthy solutions that are accessible in the communities of viewers. 

This paper focused on changes in KAB scores among those who experienced school bullying. Future studies may examine the effect of K-drama on changes in KAB scores for both perpetrators and witnesses to school bullying. Future research may further explore the relative roles of depression and anxiety on the changes of KAB scores.

## 5. Conclusions

This study demonstrates the promise of using K-dramas as an innovative social marketing tool to educate communities about mental health and school bullying. Specifically, the post-test scores revealed significant differences in knowledge among those who reported experience with and without being a victim of school bullying, and in behaviors among those who reported victim of school bullying experience. Participants reported that they “love” the drama, felt an emotional connection, and thought that K-dramas can be used as a health educational tool for Asian Americans. 

Future research may consider exploring using K-dramas to destigmatize mental health and help-seeking related to school bullying experiences (e.g., victims; perpetrators; witnesses) among college students. This study’s findings along with the aforementioned review paper on school bullying among college students point to the significant need for appropriate bullying interventions and programs for college students [18]. The use of K-dramas as a health educational tool for bullying prevention may also be developed and evaluated among other demographics such as youth. The potential implications of such future research may include reducing bullying behaviors as well as the adverse mental health effects associated with bullying. 

In conclusion, an existing community accepted approach, such as K-dramas, may be used to support bullying prevention and mental health. Such an approach is responsive to the call for community anti-bullying programs for Asian American students that was highlighted in the AAPI (Asian Americans and Pacific Islanders) Bullying Prevention Task Force Report, which included members from across the federal government, including from the White House Initiative on Asian Americans and Pacific Islanders, the U.S. Department of Justice, the U.S. Department of Education, and the U.S. Department of Health and Human Services [49].

## Figures and Tables

**Table 1:** Sample socio-demographic characteristics, by victim of school bullying experience (*n* = 118).

Characteristics	Total (*n*, %)	Victim of School Bullying		*X^2^*	*p* Value
		Yes (*n*, %)	No (*n*, %)		
Overall Gender	118, 100%	53, 44.9%	65, 55.1%		
Female Male	99, 83.9% 19, 16.1%	41, 41.4% 12, 63.2%	58, 58.6% 7, 36.8%	3.04	0.081
Current marital status					
Married Single/not married	3, 2.5% 115, 97.5%	1, 33.3% 52, 45.2%	2, 66.7% 63, 54.8%	*	1.000
Nativity					
U.S.-born Foreign-born	85, 72.0% 33, 28.0%	36, 42.3% 17, 51.5%	49, 57.7% 16, 48.5%	0.80	0.369
Employment status					
Full-time/Part-time Not employed	84, 71.2% 34, 28.8%	38, 45.2% 15, 44.1%	46, 54.8% 19, 55.9%	0.01	0.912
Ethnicity					
Non-Hispanic Hispanic Not reported/Unknown	77, 65.3% 21, 17.8% 20, 16.9%	38, 49.4% 10, 47.6% 5, 25.0%	39, 50.6% 11, 52.4% 15, 75.0%	*	0.157
Race					
Asian/Asian American Other *** Not reported/Unknown	91, 77.1% 7, 5.9% 20, 17.0%	44, 48.4% 4, 57.1% 5, 25.0%	47, 51.6% 3, 42.9% 15, 75.0%	*	0.123
Fluency in another language					
Speak only English	19, 16.1%	5, 26.3%	14, 73.7%	*	0.176
Speak some	18, 15.3%	11, 61.1%	7, 38.9%		
Speak and read	13, 11.0%	7, 53.9%	6, 46.1%		
Speak, read, and write	68, 57.6%	30, 44.1%	38, 55.9%		
Age (in years)					
Mean (SD)	22.1 (1.58)	22.0 (1.56)	22.2 (1.61)	**	0.362
Range	19–25	20–25	19–25		

* Fisher’s exact test does not have a “test statistic”; ** Two independent sample T-test, t = 0.92; *** Other races include Black or African American (*n* = 1), White or Caucasian (*n* = 2), and other, not specified (*n* = 4).

**Table 2:** Mental health and school bullying, by gender (*n* = 118).

Characteristics	Total (*n*, %)	Female (*n*, %)	Male (*n*, %)	*X^2^*	*p* Value
Had experience with depression					
Yes No Not sure	45, 38.1% 55, 46.6% 18, 15.3%	36, 36.3% 47, 47.5% 16, 16.2%	9, 47.4% 8, 42.1% 2, 10.5%	*	0.703
Presence of depressive symptoms **					
Yes (CES-D score ≥ 16) No (CES-D score < 16)	51, 43.2% 67, 56.8%	47, 47.5% 52, 52.5%	4, 21.1% 15, 78.9%	*	0.043
Had experience with anxiety					
Yes No Not sure	80, 67.8% 27, 22.9% 11, 9.3%	70, 70.7% 19, 19.2% 10, 10.1%	10, 52.6% 8, 42.1% 1, 5.3%	*	0.118
Was a perpetrator of school bullying					
Yes No Not sure	14, 11.9% 93, 78.8% 11, 9.3%	9, 9.1% 83, 83.8% 7, 7.1%	5, 26.3% 10, 52.6% 4, 21.1%	*	0.008
Witnessed school bullying					
Yes No Not sure	90, 76.3% 20,16.9% 8, 6.8%	73, 73.7% 20, 20.2% 6, 6.1%	17, 89.5% 0, 0.0% 2, 10.5%	*	0.058

* Fisher’s exact test does not have a “test statistic”; ** Center for Epidemiologic Studies-Depression (CES-D) measures presence of depressive symptoms in the past week. A score of 16 or greater is an indication of depression.

**Table 3:** Comparison of Pre- and Post-test Composite Mean Scores on Knowledge, Attitudes and Behaviors (KAB) of School-Bullying, by School Bullying Experiences (*N* = 118).

KAB	Total Mean (SD)	Victim of School Bullying		Witnessed School Bullying		Was a Perpetrator of School Bullying	
		Yes	No	Yes	No	Yes	No
		Mean (SD)	Mean (SD)	Mean (SD)	Mean (SD)	Mean (SD)	Mean (SD)
Knowledge		t = 2.86, *p* = 0.006	t = 2.96, *p* = 0.004	t = 3.11, *p* = 0.003	t = 2.41, *p* = 0.026	t = 0.54, *p* = 0.596	t = 4.17, *p* = 0.000
Pretest Posttest	4.41 (0.39) 4.53 (0.35)	4.44 (0.42) 4.58 (0.35)	4.39 (0.37) 4.49 (0.34)	4.43 (0.38) 4.54 (0.37)	4.35 (0.32) 4.50 (0.28)	4.41 (0.32) 4.46 (0.47)	4.41 (0.42) 4.54 (0.38)
Attitudes		t = 1.55, *p* = 0.127	t = 1.79, *p* = 0.078	t = 1.55, *p* = 0.126	t = 2.79, *p* = 0.012	t = −0.56, *p* = 0.584	t = 3.82, *p* = 0.000
Pretest Posttest	3.87 (0.32) 3.94 (0.33)	3.91 (0.36) 3.99 (0.34)	3.84 (0.29) 3.89 (0.32)	3.89 (0.33) 3.94 (0.34)	3.84 (0.30) 3.96 (0.30)	3.94 (0.38) 3.88 (0.48)	3.86 (0.32) 3.96 (0.31)
Behaviors		t = 3.09, *p* = 0.003	t = 0.34, *p* = 0.738	t = 1.27, *p* = 0.207	t = 1.40, *p* = 0.178	t = 0.64, *p* = 0.535	t = 1.71, *p* = 0.091
Pretest Posttest	3.48 (0.49) 3.54 (0.45)	3.49 (0.50) 3.67 (0.42)	3.45 (0.49) 3.44 (0.45)	3.52 (0.49) 3.58 (0.47)	3.29 (0.49) 3.41 (0.38)	3.57 (0.59) 3.66 (0.48)	3.43 (0.48) 3.51 (0.46)

There were 12 knowledge, 20 attitudes and 8 behaviors items.

**Table 4:** Comparison of Pre- and Post-test Composite Mean Scores on Knowledge, Attitudes and Behaviors (KAB) of School-Bullying, by Mental Health Issues (*N* = 118).

KAB	Total Mean (SD)	Had Experience with Depression		Presence of Depressive Symptoms		Had experience with Anxiety	
		Yes	No	Yes	No	Yes	No
		Mean (SD)	Mean (SD)	Mean (SD)	Mean (SD)	Mean (SD)	Mean (SD)
Knowledge		t = 4.22, *p* = 0.000	t = 2.07, *p* = 0.043	t = 2.83, *p* = 0.006	t = 3.01, *p* = 0.004	t = 3.87, *p* = 0.000	t = 1.30, *p* = 0.207
Pretest Posttest	4.41 (0.39) 4.53 (0.35)	4.37 (0.44) 4.53 (0.35)	4.41 (0.05) 4.52 (0.05)	4.41 (0.38) 4.52 (0.34)	4.41 (0.40) 4.54 (0.36)	4.41 (0.39) 4.53 (0.37)	4.43 (0.40) 4.52 (0.33)
Attitudes		t = 1.03, *p* = 0.311	t = 1.48, *p* = 0.144	t = 2.08, *p* = 0.042	t = 1.20, *p* = 0.234	t = 2.78, *p* = 0.007	t = −0.03, *p* = 0.974
Pretest Posttest	3.87 (0.32) 3.94 (0.33)	3.87 (0.35) 3.92 (0.34)	3.89 (0.32) 3.96 (0.34)	3.84 (0.30) 3.93 (0.32)	3.90 (0.34) 3.95 (0.34)	3.83 (0.32) 3.93 (0.35)	4.04 (0.28) 4.04 (0.29)
Behaviors		t = 2.03, *p* = 0.00	t = 0.08, *p* = 0.934	t = 2.53, *p* = 0.015	t = 0.41, *p* = 0.680	t = 2.92, *p* = 0.005	t = −0.86, *p* = 0.397
Pretest Posttest	3.48 (0.49) 3.54 (0.47)	3.46 (0.51) 3.57 (0.48)	3.49 (0.51) 3.51 (0.48)	3.30 (0.46) 3.44 (0.38)	3.60 (0.47) 3.62 (0.49)	3.37 (0.45) 3.51 (0.47)	3.76 (0.55) 3.69 (0.42)

There were 12 knowledge, 20 attitudes and 8 behaviors item.

## Appendix A

**Table A1:** Knowledge, attitudes and behaviors composite scores.

**Knowledge (12 Items)**	**Source**
1. School bullying victims are at risk for being depressed.	A
2. School bullying victims are at risk for being suicidal.	A
3. Bystanders are considered “innocent.” (r)	C
4. Bullying is defined as the use of one’s strength or popularity to injure, threaten, or embarrass another person on purpose.	A
5. Bullying can be physical, verbal, or social.	A
6. You have prior knowledge on school bullying.	*
7. Bullying occurs inside school as well as outside of school.	C
8. There is a link between bullying behavior and performance in school.	A
9. Being bullied takes a toll on one’s health.	A
10. Persons who bully others are likely to drop out of school.	C
11. Bullying is a form of violence.	C
12. Bullying should be stopped because it affects social, emotional, and mental well-being.	C
**Attitude (20 Items)**	
1. I know what school bullying is.	A
2. You feel that the issues on school bullying are not exposed, publicized enough, or at all at your university.	C
3. School bullying is a problem at your university.	A
4. You are aware of the resources offered at your university regarding school bullying.	*
5. If I am a victim of being bullied, I would feel comfortable speaking up to my friend.	A
6. If I am a victim of being bullied, I would feel comfortable speaking up to my parents.	A/C
7. If I am a victim of being bullied, I would feel comfortable speaking up to my teachers.	A/C
8. If I am a victim of being bullied, I would feel comfortable speaking up to my counselors.	A
9. School bullying is acceptable depending on the circumstance. (r)	A
10. School bullying is never acceptable.	C
11. I feel threatened by other students. (r)	A/C
12. You are aware of the other different forms of bullying such as cyber bullying, racial bullying, and sexual bullying.	C
13. You believe that bullies have a strong need for power and dominance over others. (r)	C
14. I want to help address the issue on school bullying.	C
15. I feel that students need to be more informed on school bullying.	A
16. Your university should have a school-bullying curriculum.	A
17. Teachers should interfere if they see a bullying in act.	C
18. You believe those who bully should be punished.	C
19. You believe those who bully should receive help from teachers/administrators.	C
20. Students that are bullied usually deserve it. (r)	R
**Behaviors (8 Items)**	
1. If I am a victim of being bullied, I do not want anyone to know. (r)	A
2. If I am a victim of being bullied, I know I am capable of handling it myself.	A
3. If I saw my peer being bullied, I would immediately interfere/stop it.	C
4. I would be able to confront the person bullying me.	C
5. I will not be judged if I share that someone is bullying me.	C
6. If a bully gets physical with you, you will use physical self-defense to protect yourself. (r)	C
7. I would give money or other incentives to the bully to stop harassing me. (r)	C
8. I know at least one teacher/administrator who I can seek help from.	C

Abbreviations: “A”—Authoritative School Climate Survey; “C”—Centers for Disease Control and Prevention (CDC) Measuring Bullying Victimization, Perpetration, and Bystander Experiences: Compendium of Assessment Tools; “R”—Revised Pro-victim Scale; Notes: (r) reverse coded; * This item was developed for the purpose of the study.
